# Supplementary material for: Macrogenomic and Metabolomic Analyses Reveal Mechanisms of Gut Microbiota and Microbial Metabolites in Diarrhea of Weaned Piglets
Source: Animals (Basel). 2024 Aug 12;14(16):2327. doi: 10.3390/ani14162327 (PMC11350701; doi:10.3390/ani14162327)
Supplement: Supplementary file 1 [file animals-14-02327-s001.zip › animals-3107812-supplementary.pdf]

A detailed supplement of the method

Supplementary methods S1.

S1. Metagenomic analysis

Fastp (version 0.20.0)[1] and Burrows-Wheeler Aligner (BWA) (version 0.7.9a)[2] software were used to assess the quality of the raw data, and splicing assembly was performed using MEGAHIT (version 1.1.2)[3] software and the contigs with sequence fragment size >300 bp were used as the final assembly results. Next, contigs with sequence fragment size >300 bp were subjected to gene prediction by MetaGene [4] (http://metagene.cb.k.u-tokyo.ac.jp/). The predicted gene sequences were clustered using the software CD-HIT (version 4.6.1)[5] with parameters set to 90% identity and 90% coverage and the longest genes were screened as representative sequences to construct a non-redundant gene set. Finally, SOAPaligner (version 2.21)[6] software was used to compare the high-quality reads in the samples with the non-redundant gene set (parameter setting: 95% identity) to obtain the data of gene abundance in the corresponding samples. The non-redundant gene sets were compared with the National Center for Biotechnology Information (NCBI) non-redundant (NR) database using Diamond (version 0.8.35)[7] software (parameter set with an expected e-value of 1×10-5). Species annotations were obtained and their abundance was calculated and compared against the Kyoto Encyclopedia of Genes and Genomes (KEGG) database [8]. Subsequently, the corresponding KEGG functions were obtained and the abundance of the corresponding functional categories was calculated.

Gut microbial alpha diversity including Chao 1, Shannon, and Simpson indexes, was analyzed using the Mothur (version 1.30.2) software, and partial least squares discriminant analysis (PLS-DA) was used to assess gut microbial beta diversity. Differences in microbial composition and function between weaned healthy and diarrheic piglets were assessed by analysis of similarities (ANOSIM) and linear discriminant analysis effect size (LEfSe; version 1.1.2)[9]. Data were visualized and statistically analyzed using R software (version 3.3.1).

[1] Chen S , Zhou Y , Chen Y ,et al.fastp: an ultra-fast all-in-one FASTQ preprocessor [J].Bioinformatics, 2018, 34(17):i884-i890.

[2] Li H , Durbin R .Fast and accurate short read alignment with Burrows-Wheeler transf orm[J].Bioinformatics (Oxford, England), 2009, 25(14):1754-60.

[3] Dinghua L , Chi-Man L , Ruibang L ,et al.MEGAHIT: an ultra-fast single-node soluti on for large and complex metagenomics assembly via succinct de Bruijn graph[J].Bioi nformatics, 2015, 31(10):1674-1676.

[4] Hideki N , Jungho P , Toshihisa T .MetaGene: prokaryotic gene finding from environ mental genome shotgun sequences[J].Nucleic Acids Research, 2006, 34(19):5623-5630.

[5] Fu L , Niu B , Zhu Z ,et al.CD-HIT: accelerated for clustering the next-generation se quencing data[J].Bioinformatics (Oxford, England), 2012, 28(23):3150-2.

[6] Li R , Li Y , Kristiansen K ,et al.SOAP: short oligonucleotide alignment program[J]. Bioinformatics, 2008, 24(5):713-714.

[7] Huson D H , Buchfink B .Fast and sensitive protein alignment using DIAMOND[J].N ature Methods, 2015.

[8] Ogata H , Goto S , Sato K ,et al.KEGG: kyoto Encyclopedia of Genes and Genomes [J].Nucleic Acids Research, 1999, 27(1):29-34.

[9] Fang, Chang,Shishi, He,Chenyuan, Dang,Assisted Selection of Biomarkers by Linear D

iscriminant Analysis Effect Size (LEfSe) in Microbiome Data.[J] .J Vis Exp, 2022, 0: 0.

Supplementary methods S2.

S2.LC–MS Untargeted Metabolomics Analysis

After the samples were thawed (-4°C), 100-µL of sample was aspirated and transferred into an Eppendorf tube, 400 µL of extraction solution consisting of a 1:1 ratio of acetonitrile and methanol was added, vortexed, and mixed (30S), and then low-temperature ultrasonic extraction was performed (5 °C, 90 min, 40 KHz). Next, the samples were left to stand (-20 °C, 30 min), then centrifuged (4 ℃, 15 min, 13,000 g), and the supernatant was pipetted. The supernatant was blown dry under nitrogen, and then added with 100 µL of acetonitrile and water at a ratio of 1:1, low-temperature ultrasonic extraction (5°C, 5 min, 40 KHz) was performed, followed by centrifugation (4℃, 5 min, 13000 g), and the supernatant obtained afterward was used for quality assessment and online analysis.

The raw data were processed using Progenesis QI (Waters Corporation, Milford, MA, USA) software for baseline filtering, peak identification, and peak alignment, and the mass spectrometry (MS) and tandem mass spectrometry (MS-MS) spectral information was matched with HMDB (http://www.hmdb.ca/) and Metlin (http://metlin.scripps. edu/) resources to obtain metabolite annotations. PLS-DA and orthogonal partial least squares discriminant analysis (OPLS-DA) were performed using the R software package “ropls” (version 1.6.2), and model stability was assessed using seven cycles of cross-validation. In addition, Student's t-tests and multiplicative analysis of variance were performed. The selection of differential metabolites was determined based on the variable importance in the projection (VIP) obtained from the OPLS-DA model and the Student's t-test P-value, and the metabolites with VIP>1, P<0.05 were considered as differential metabolites. Differential metabolites were obtained by metabolic pathway annotation using the KEGG database to identify the pathways in which the differential metabolites were involved.

Supplement Tables S1:

Table S1: The top 35 significantly changed metabolites[1]

Metabolites	FX	JK	VIP	Fold change	*P*-value	Up/down
**N-Valylphenylalanine**	4.894	3.691	4.116	1.326	0.001	up
**Norepinephrine**	4.673	3.482	3.833	1.342	0.012	up
**Pelargonidin 3-sophoroside**	4.594	5.864	3.742	0.783	0.050	down
**2-Hydroxypyridine**	4.059	2.823	3.634	1.438	0.003	up
**Adipostatin A**	4.424	5.406	3.301	0.818	0.001	down
**8-Azaspiro[4.5]decane-7,9-dione**	3.935	3.010	3.308	1.307	0.021	up
**Sulfuric acid 4-methoxyphenyl ester**	5.487	4.413	3.114	1.243	0.025	up
**Deoxynivalenol**	4.353	5.135	2.939	0.848	0.043	down
**Phaseolic acid**	3.338	4.142	2.585	0.806	0.045	down
**Melanettin**	4.281	3.566	2.835	1.201	0.044	up
**N(epsilon)-(Carboxymethyl)hydroxylysine**	3.055	3.740	2.784	0.817	0.029	down
**Adenosine 2'-phosphate**	4.229	4.808	2.712	0.880	0.008	down
**Spongothymidine**	5.137	4.647	2.536	1.105	0.006	up
**Carnosine**	4.517	5.056	2.465	0.893	0.001	down
**Lysyl-Glycine**	4.874	4.345	2.541	1.122	0.022	up
**Devd-amc**	4.736	4.114	2.298	1.151	0.039	up
**Methylimidazoleacetic acid**	5.654	5.257	2.425	1.076	0.000	up
**Sporol**	4.429	4.924	2.332	0.900	0.037	down
**Methyldopa**	4.290	3.876	2.194	1.107	0.023	up
**Indolelactic acid**	5.319	4.919	2.292	1.081	0.004	up
**Isobutyl 4-hydroxybenzoate**	4.130	4.614	2.108	0.895	0.019	down
**Ascorbic Acid**	5.184	4.699	2.062	1.103	0.030	up
**Gynocardin**	5.383	5.806	2.060	0.927	0.010	down
**Pyridoxal**	4.544	4.905	2.138	0.926	0.008	down
**7-Epijasmonic acid**	5.385	5.032	2.018	1.070	0.000	up
**3-hydroxydecanoyl carnitine**	4.870	5.203	2.154	0.936	0.009	down
**Tulathromycin A**	6.822	6.505	1.790	1.049	0.009	up
**Deoxyuridine**	5.105	5.390	1.805	0.947	0.001	down
**(3S)-3-hydroxycyclocitral**	4.995	5.285	1.687	0.945	0.012	down
**7-Aminomethyl-7-carbaguanine**	4.412	4.658	1.651	0.947	0.042	down
**2-Hydroxycampholonic acid**	4.943	5.225	1.542	0.946	0.041	down
**Parasorbic acid**	4.365	4.601	1.437	0.949	0.031	down
**Indole-3-Carboxaldehyde**	6.177	5.980	1.472	1.033	0.038	up
**Indole-3-acetaldehyde**	5.977	5.763	1.403	1.037	0.019	up
**Carboxycelecoxib**	5.309	5.101	1.319	1.041	0.044	up

[1]FX= weaned diarrhoeic piglets, JK = weaned healthy piglets
